# “Your struggles are valid, you are worthy of help and you deserve to recover”: narratives of recovery from orthorexia nervosa

**DOI:** 10.1007/s40519-023-01554-3

**Published:** 2023-02-27

**Authors:** Catherine V. Talbot, Charleigh E. R. Campbell, Maddy Greville-Harris

**Affiliations:** Department of Psychology, Bournemouth University, Poole House, Fern Barrow, Poole, BH12 5BB Dorset UK

**Keywords:** Feeding and eating disorders, Eating behaviour, Online communities, Disordered eating, Healthy eating

## Abstract

**Purpose:**

Limited research has examined recovery processes and conceptualisations of recovery within orthorexia nervosa (ON). This study harnessed Instagram data to examine how people who self-identify with ON use the hashtag #OrthorexiaRecovery and how recovery is represented within this online space.

**Methods:**

500 textual posts containing #OrthorexiaRecovery were extracted from Instagram. Co-occurring hashtags were analysed descriptively to determine whether this online space is specific to ON, and textual data were analysed using reflexive thematic analysis.

**Results:**

The hashtag analysis indicated that #OrthorexiaRecovery is being used within a wider context of eating disorder recovery and awareness, but also provides deep insights into experiences of recovery from ON. The thematic analysis generated five themes: the invisibility of orthorexia; a turbulent and continuous process; finding food freedom; from compulsive exercise to intuitive movement; a community of support. Our findings suggest that people with self-reported ON experience recovery as a continuous process and the current invisibility of ON within diagnostic criteria and wider society impedes recovery. While working towards recovery, users aimed to be free from diet culture, become more attuned to their bodies, and develop more adaptive relationships with food and fitness. Users noted a general lack of support for people with ON and so used this online space to create a supportive community, though some content was potentially triggering.

**Conclusion:**

Our findings highlight the importance of increased recognition of ON and the potential value of targeting societal norms and harnessing social identity resources within therapeutic interventions for ON.

## Introduction

Orthorexia nervosa (ON) is characterised by an obsession with healthy eating that is extreme and pathological [1]. In ON, fixation is on food quality or purity rather than quantity, with the aim of maximising one’s health and wellbeing [2, 3]. Despite aiming to boost health, ON has been associated with impairments in wellbeing [4], involving complex and often concerning conceptualisations of what constitutes ‘health’ and ‘healthy eating’ [5]. By refining food consumption to a restricted number of ‘clean’ or ‘healthy’ foods, those with ON are at risk of facing adverse outcomes such as malnutrition, significant weight loss, psychological distress, and damaged social wellbeing [2, 6]. While ON is not currently classified as a disorder, several sets of diagnostic criteria have been proposed with a general consensus that ON involves: (1) an obsessional preoccupation with ‘healthy’ or ‘clean’ foods; (2) rigid avoidance of food considered to be ‘unhealthy’ or ‘unclean’; (3) distress when ‘food rules’ are violated; (4) social, physical, and/or psychological wellbeing impairments because of these beliefs and behaviours [6–8]. Given that ON is a proposed ‘new’ eating disorder, there is still much to learn about its development, maintenance, and recovery processes.

Understandings of recovery processes and how recovery is conceptualised within the context of ON is currently lacking, likely due to ON not being an officially recognised eating disorder within official diagnostic criteria [6–8]. Preliminary research in this area suggests that self-reflection is an important first step in recovery, through which individuals with ON learn to accept their behaviours as disordered and begin their journey of recovery [9]. In an online ethnography, Ross Arguedas [10] found that shifting identities is central to recovery from ON. The changes that individuals experienced during recovery were associated with a sense of marginalisation or exclusion from health-oriented cultures and were left questioning who they are. However, through self-identification with ON, individuals were able to reframe health-obsessive past identities as ‘pathological’ rather than idealistic, and position changing identities as ‘healing’. People with experience of ON have also discussed the continuous nature of recovery, noting their enduring anxieties around food despite the severity of the disorder having drastically improved [11]. Nevertheless, efforts towards recovery from ON appear to come with greater social freedom, increased happiness, and lessened anxiety [9, 11].

The role of social media in the development and maintenance of other eating disorders has been well explored [12–14]. Emerging research on ON has emphasised the triggering nature of social media content and its role in developing ON [15–17]. For example, researchers have noted the dominance of clean eating content on Instagram [18, 19], and have found that Instagram usage is associated with elevated levels of ON symptoms [20]. However, the relationship between social media and ON—as well as other eating disorders—is far more complex, with research showing that different types of engagement with social media, frequency of usage, and reasons for usage are important factors in eating disorder symptomology [21–23]. Importantly, social media can also provide valuable recovery spaces for people with ON [24–26]. For example, Valente et al. [24] reported that while social media can fuel ON behaviours, the spaces afforded by social media can also be valuable stimuli for recovery, providing a counterculture to mainstream sociocultural ideals of health and beauty through the promotion of body positivity and intuitive eating.

As people who self-identify as having ON are using social media in this way, researchers have examined online data to gain insight into lived experiences [10, 23, 27], particularly through the analysis of Instagram data [28]. It is important to note that those who write about these experiences on social media either self-identify as having ON or have received some other medical diagnosis, due to ON not currently being recognised as a medical diagnosis [6–8]. However, as noted by Valente et al. [24], research that engages with lived experiences can provide vital experiential knowledge to develop clinical understandings of and approaches to ON. Consistent with this, researchers have examined discussions of ON by analysing the hashtag ‘#Orthorexia’, finding that Instagram encourages problem realisation and a sense of belonging through this hashtag [25]. In an analysis of this hashtag, Santarossa et al. [23] found that the ON Instagram community is a relatively small but supportive community which focuses on recovery and adopting healthier eating behaviours. However, they found some images were associated with specific diets (e.g., veganism, paleo), indicating that some content may promote adherence to restrictive eating behaviours.

While some of these researchers have included recovery hashtags (i.e. #OrthorexiaRecovery) in their data collection strategies [24, 25], internet-mediated research that specifically focuses on recovery is limited [10]. We address this gap in the literature, building upon the work of Valente et al. [24, 25] and Ross Arguedas [10] to examine narratives of recovery under the hashtag ‘#OrthorexiaRecovery’. At the time of writing, there are over 121,000 Instagram posts with the hashtag ‘#OrthorexiaRecovery’, representing a previously untapped source of knowledge about recovery processes. In this research, we aimed to explore: (1) how people use the hashtag #OrthorexiaRecovery on Instagram; (2) how recovery is represented within this hashtag.

## Methods

### Sample and data collection

To extract the data, the search term ‘#OrthorexiaRecovery’ was input into Instagram’s search engine in June 2021, finding over 121,000 posts. The text pertaining to the 500 most recent posts using #OrthorexiaRecovery were copied and pasted into a Microsoft Word document by the first author. This sample size was deemed appropriate to provide the depth and breadth of data required to answer our research questions and is also in keeping with other research that utilises online data [29, 30]. Only the captions uploaded with Instagram posts were extracted for analysis; comments on individual posts were not included in this study. Images were extracted using screen-capture technology. Each post was downloaded and saved as it appeared, including original formatting. All original posts were uploaded to Instagram between May and June 2022.

### Analysis

Our analysis had two distinct phases. Firstly, a descriptive analysis of co-occurring hashtags was conducted to determine patterns of hashtag usage. Here, we define ‘co-occurrence’ as the incidence of any pair of hashtags used together in the same Instagram post, as per Wang, Liu, and Gao [31]. The purpose of this analysis was to determine whether the #OrthorexiaRecovery Instagram space was specific to ON or included other eating disorder communities. This descriptive analysis involved exporting all of the hashtags into a separate word document which was then uploaded to Nvivo12. A Word Frequency query was then run within the software to list the most frequently occurring hashtags.

During Phase Two, textual data from Instagram posts were analysed using reflexive thematic analysis [32]. Reflexive thematic analysis was the chosen method of analysis because it enabled us to meet the research objective of identifying patterns of meaning across the data. We also chose this method because it was most familiar to the research team and fit with the theoretical underpinnings of the work, in which subjectivity was viewed as an important “analytic resource” [33] and any meaning-making that resulted from the research was viewed as context-bound, positioned and situated [34].

The reflexive thematic analysis involved several stages. First, all authors began by reading a sub-sample of the posts, to support an in-depth familiarisation with the data. During this stage, images were also reviewed by the authors to provide additional context to the textual data. Preliminary codes were then generated by the first and third authors, with codes focusing on recovery processes and conceptualisations of recovery. These codes were then applied to the dataset by the first and third author, using the comments feature in Microsoft Word. Textual posts were coded in an inductive way, reflecting the explicit content of the data. Similarly coded data were then grouped together to generate initial themes. Themes were then reviewed by all authors and revised to ensure they captured the dataset appropriately. The analysis was an iterative process, which involved repeatedly moving between the raw data and generated themes. To promote transparency, a clear audit trail was kept throughout the analysis which documented theme development. Across all stages of the analysis, authors engaged in reflexive discussions, in which we reflected upon our diverse assumptions of the data and developed our analysis through nuanced debate and discussion.

### Ethics

Ethical approval was obtained from Bournemouth University Ethics Committee. The British Psychological Society [35] ethical guidelines state that unless consent has been sought, observation of public behaviour must take place in public situations where people would expect to be observed by strangers. In this respect, public online spaces can also be regarded as public behaviours. Therefore, in this study only public Instagram posts were collected and analysed. While we viewed our data as being located within the public domain, to maintain respect for account holders and promote ethical practice within research that uses online data, reported quotations were paraphrased in a manner that retains original meaning with all authors agreeing on the phrasing of each quotation. This is because a Google search of a quoted post could lead directly to the accounts from which it was posted. This approach is consistent with current recommendations and practice in internet-mediated research [36, 37].

## Results

### Hashtag analysis

Frequency analysis of co-occurring hashtags showed that hashtags relating to orthorexia nervosa frequently featured in the dataset, including #Orthorexia (*n* = 88), #IntuitiveEating (*n* = 81), #AllFoodsFit (*n* = 62), and #AntiDietCulture (*n* = 48). There was also a notable co-occurrence of hashtags relating to eating disorders more generally (e.g., #EDRecovery, *n* = 145; #EatingDisorderRecovery, *n* = 114), as well as hashtags associated with other specific eating disorders such as #BulimiaRecovery (*n* = 96) and #BingeEatingRecovery (*n* = 95). These findings indicate that the hashtag #OrthorexiaRecovery is being used within a wider context of eating disorder recovery and awareness. The 20 most frequently featured hashtags are reported in Table 1.

**Table 1:** Frequency of co-occurring hashtags

Hashtag	Count
#EdRecovery	145
#EatingDisorderRecovery	114
#BulimiaRecovery	96
#BingeEatingRecovery	95
#Orthorexia	88
#IntuitiveEating	81
#AntiDiet	78
#EatingDisorderAwareness	77
#FoodFreedom	72
#MentalHealthAwareness	70
#AnorexiaNervosaRecovery	68
#MentalHealth	63
#AllFoodsFit	62
#AnorexiaRecovery	60
#Haes*	56
#Recovery	54
#BodyPositivity	49
#AntiDietCulture	48
#HealthAtEverySize	47
#DisorderedEating	44

*Haes = health at every size

### Reflexive thematic analysis

Our reflexive thematic analysis of textual Instagram posts generated five themes: (1) the invisibility of orthorexia; (2) a turbulent and continuous process; (3) finding food freedom; (4) from compulsive exercise to intuitive movement; (5) a community of support. These themes are outlined below, using paraphrased quotations to illustrate the findings. The extracts are followed by a number which indicates their individual identifier.

### Theme 1: the invisibility of orthorexia

Users shared some of the obstacles they faced on their recovery journey, most notably the *‘invisible’* struggle of those with ON due to the normalisation of ON behaviours within western society [38]. For example, users described the difficulties associated with people with ON being perceived as healthy, which served to idealise and reinforce ON behaviours:

*The person who values health, nutrition and fitness. The person who exercises daily. The person who eats healthily. The person who’s self-control and discipline are praised. They may be the one grappling with anxiety, fear, and preoccupied with disordered thoughts. Their struggle may go unnoticed due to how our culture glorifies these behaviours *[174].

The perceived dominance of healthism discourse (i.e. the pervasiveness of health as an ideal, that places the burden of responsibility on the individual; [39]) within western society led users to feel as though ON was not taken seriously as an eating disorder. Ultimately, these users explained that not being viewed as “sick enough” represented a key barrier in their recovery journey:

*It hurts when people say my orthorexia is not serious because I don’t have anorexia. People say I have amazing control over food and ask how I’m struggling when I am “so healthy” *[115].

Users reported that ON was not only invisible to others, but also to the person experiencing it. That is, users described being unaware that their behaviours and the associated outcomes are disordered, thus delaying recognition that they were experiencing an eating disorder.

*I was the most unhealthy when I was at my lowest weight, but I was unaware that it was not okay to do what I was doing *[381].

Users also described “*exercising so much control*” in order to pursue a lifestyle which is so valued within Western society. These health behaviours were so ingrained within those experiencing ON that being ‘healthy’ was viewed as an important part of their identity; in turn, this exacerbated the invisibility of ON due to disordered health behaviours being viewed as a core and unchangeable aspect of themselves. As such, this presented a barrier to recovery as users felt it was equivalent to changing who they are as a person.

*I was sure I would always live a healthy lifestyle. It was part of me. I didn’t think my personality could change* [113].

### Theme 2: a turbulent and continuous process

Users tended to position their journey to recovery as a turbulent process with many setbacks. For example, users described dealing with a resurgence of invasive thoughts and negative emotions around eating certain foods. These users emphasised that recovery was non-linear, and one person used the metaphor of a “‘*roller coaster*” to describe the recovery process. Consequently, users encouraged others to celebrate the “*small steps towards recovery and away from ED behaviours*”, while others highlighted that setbacks were a part of the recovery journey:

*Truthfully, recovery is not easy, and I still struggle. I have good days and bad days. There are days when I feel guilty for eating, want to avoid snacking, and it’s hard to finish my dinner. There are days when I feel compelled to exercise and I feel terrible in my body *[200]*.*

Users explained that recovery was a highly personal and ongoing process, taking much time and commitment. Under the #OrthorexiaRecovery hashtag, users expressed that there was no “*finish line*” in recovery, instead identifying recovery as a gradual and enduring process.

*Recovery is a continual process of choosing yourself over fear, rather than a finishing line that can be crossed*  [35].

In some cases, discussions of recovery from ON were intertwined with other eating disorders. For example, one user described their journey of recovering from anorexia nervosa but then experiencing ON later in life, recurrently moving between recovery and relapse. These users tended to position recovery as “*never a one-time thing*”. Instead, while amid recovery and relapse, recovery was framed as an individual choice which must be actively made each day.

*I was living with anorexia at 14 years old, which I recovered from by the end of high school. A couple of years later, I started living with orthorexia on and off for eight years. I’ve now recovered and no longer have an eating disorder. I have relapsed but that does not mean I’m a failure. Recovery is not a one-time accomplishment, but rather something you chose for yourself everyday* [493]*.*

### Theme 3: finding food freedom

Users regularly used #OrthorexiaRecovery to reject messages rooted in diet culture (i.e. pervasive cultural norms that emphasise thinness, control, and food restriction, as well as the moralisation of food; [40]), moving towards a more positive attitude to food. A shared perception among users of #OrthorexiaRecovery was that diet culture reinforces harmful ideals and normalises maladaptive behaviours and mindsets, thus distorting conceptualisations of health.

*The messages of diet culture have altered many people’s value of health into something that is not healthy at all* [446].

Users felt this distorted perception of health contributed to the development and maintenance of ON behaviours. Therefore, users emphasised the importance of distancing oneself from and actively ‘unlearning’ the principles of diet culture to facilitate recovery. For example, users referred to experiences through which they learned to eat certain food that had been demonised by diet culture, by granting themselves (and others) permission to eat and recognising that eating is not something which needs justification.

*You are allowed to enjoy food when you are not hungry or are beyond satisfied. You are allowed to spontaneously eat out with friends and family. You are allowed to eat more than usual during holidays, or simply because you want to!* [71].

The value of ‘intuitive eating’ was frequently discussed under #OrthorexiaRecovery; an adaptive eating style which involves listening to the body and positioning it as intrinsically knowing the quantity and type of food to eat [41]. Consistent with this, users rejected food restriction and instead accepted that *“all foods fit*” within a balanced diet. That is, specific foods were viewed as neither good nor bad.

*Mac n Cheese used to be a big no because of its carbs and dairy contents. If I ate Mac n Cheese in the past, I would feel sick with guilt, anxiety and shame. To counteract meals like this that I considered “dirty” or “cheat” meals, I would cut calories and do HIIT every day for the next week to “burn it off”. In recovery, I am headed towards food freedom, intuitive eating and intuitive movement. So, this is more than just Mac n Cheese; this symbolises a massive step toward recovery *[4].

This shift in attitudes towards food was believed to have several psychological benefits, including increased self-compassion, confidence, and emotional wellbeing. Users also reported lessened anxiety and guilt about food, being able to enjoy food again without being burdened by its nutritional value.

*I have fallen in love with myself again after fixing my relationship with food. Food consumption is of course vital, but our relationship with food is far more important *[207]*.*

*When I visited home, my mum baked a flapjack. It contained golden syrup and butter, and I was fine with that. I didn’t scrutinise the shape and size of each piece. Instead, I ate several pieces and enjoyed every bite!!* [132].

### Theme 4: from compulsive exercise to intuitive movement

Our fourth theme highlights how account holders viewed their relationship with fitness as a core component of recovery, despite it not being a core component of proposed ON diagnostic criteria [6–8]. These users often reflected upon their problematic attitudes towards fitness when they were struggling with ON, making explicit reference to engaging in excessive exercise behaviours, often with the aim of weight loss:

*I hated the treadmill, yet I was addicted to it [179]. *

*The only purpose the gym had was to make my body smaller [379]. *

When reflecting on these experiences, some users noted the impact that fitness subcultures on social media had on them, notably fitspiration content (content which ostensibly aims to inspire fitness [30]). However, some users explained that a viewer’s mindset is what makes fitspiration harmful, rather than the content itself. Therefore, for these users a shift in attitudes toward fitness and exercise appeared to be an important element of recovery.

*Fitspiration itself did not hurt me, my interpretation and response to it did [181].*

Indeed, users turned to #OrthorexiaRecovery to challenge myths surrounding fitness, combatting maladaptive attitudes towards health among those viewing the hashtag. For example, users challenged the idea that a toned body is a healthy body and offered re-assurance about the importance of rest.

*It is a myth that appearing toned is a sign of strength or health. Would you not rather feel strong than only look it? [359].*

*It’s okay to give your body time to heal. Your progress will not be ruined by a week or even a month of rest, and who cares if you gain weight! [495].*

One key way in which users developed a healthier relationship with exercise was through ‘intuitive exercise’, which involves listening to the body for cues about exercise. Through listening to their bodily cues, users explained that the need to exercise everyday was lessened and, in turn, feelings of shame and anxiety were reduced.

*Intuitive movement can completely remove the anxiety and shame that comes with choosing rest over exercise [209].*

### Theme 5: a community of support

The importance of support from others on the path to recovery was often discussed by users, with support being positioned as a valuable ‘aid’ to recovery. However, some users noted that ON not being recognised as a clinical diagnosis created barriers to accessing support (see Theme 1). Therefore, these users turned to #OrthorexiaRecovery to provide validation and encouragement for those struggling with ON.

*Don’t hesitate to reach out if you are struggling. Even though there is still no official diagnosis for orthorexia, your struggles are valid, you are worthy of help and you deserve to recover [143].*

Given this challenge in accessing support, the #OrthorexiaRecovery community on Instagram appeared to be a particularly supportive space where users could provide encouragement, express solidarity, and inspire hope that recovery is possible.

*The purpose of this account is to share my eating disorder recovery with the hope of helping and educating others, as similar accounts helped me through my own recovery. I will hold myself accountable by sharing my overall progress, including wins and losses [267].*

There was variation in the types of support which users provided within this online space. Emotional support that provided messages of empathy or encouragement were common, as well as informational support messages that signposted users to helplines and network support that linked users with virtual recovery support groups.

*Continue making choices that are uncomfortable to move towards recovery and true health. Yes, it is hard, but you can do it! < 3 [14].*

*Join our free online support group to help you progress with your recovery journey this Thursday evening on Zoom! [330].*

Other users provided more practical support, by sharing meal recipes that they perceived as useful in recovery. Despite this support, we observed some nutritional content which could be triggering for those experiencing ON, such as users promoting restrictive diet choices under the hashtag.

*We are sharing a warming, delicious, and nourishing lentil soup recipe as we transition into Winter. As well as being an easy recipe, this soup is plant-based and contains energy that is essential for recovery [21].*

*For months now, I have solely used side dishes to plate my good. For lazy days, I keep Kodiak products (protein) but usually throw them out as they either freezer burn or go out of date. They are a fun chocolate treat while experiencing PMS [153].*

## Discussion

In this study, we investigated narratives of recovery from ON through an analysis of the hashtag ‘#OrthorexiaRecovery’. Our findings suggest that people with self-identified ON experience recovery as a continuous process with many setbacks, and the current invisibility of ON within diagnostic criteria and wider society impedes recovery processes. While working towards recovery, people with ON aimed to be free from food rules that are rooted in diet culture, become more attuned to their bodies, and develop more adaptive relationships with food and fitness. Users noted a lack of support for people with ON and so used this online space to create a community of support, though some content was observed to be potentially triggering for those experiencing or at risk of ON.

A crucial barrier to recovery from ON was its invisibility at both a societal-level and within formal diagnostic criteria. This finding is consistent with other research, in which people with ON have reported a widespread lack of awareness, resulting in confusion and delegitimisation of disordered eating [26, 27]. A lack of recognition of ON at a diagnostic- and societal-level may ultimately cause it to go undetected and under-reported, thus potentially exacerbating severe medical consequences, psychological distress, and impairments in other important areas of functioning [6, 8]; whereas better recognition of ON may lead to improved access to and quality of treatment. Better awareness of ON within society could be achieved by incorporating it in eating disorder awareness, education, and prevention programmes, which have had some success for other eating disorders [42, 43].

In terms of the clinical implications for this study, the widespread invisibility of ON further emphasises the potential need for increased awareness of the proposed diagnostic criteria and symptoms of ON [8] particularly within healthcare, eating disorder and mental health settings. Failure to recognise ED illness severity is as a barrier to treatment seeking more generally [44]; our participants reported similar difficulties with recognising and accessing support for their ON symptoms. Furthermore, while there may be barriers to treatment seeking, no evidence-based treatment programme exists at present to specifically target the symptoms of ON [45]. While this study reflects on some positives of online recovery spaces, it also highlights some potential areas for further research in ON treatment; specifically in targeting culturally reinforced maladaptive mindsets and behaviours related to ‘healthy’ eating and exercise.

Users of #OrthorexiaRecovery were particularly critical of beliefs that were perceived to be rooted in diet culture, for example the idea that there are ‘bad’ foods that do not fit within a healthy and balanced diet, and the belief that food consumption should not be enjoyable. Similar concerning conceptualisations of ‘health’ have been identified in a sample of people at risk of ON, in which respondents emphasised the importance of food being functional rather than enjoyable and described the dangers of ‘wrong’ foods [5]. Users in our study also noted the importance of ‘shifting identities’ during recovery, mirroring the findings of Ross Arguedas [10] who identified that people with ON experience identity-conflict due to exclusion from health-oriented cultures. These findings have implications for future therapeutic interventions for ON, which may be successful in harnessing social identity resources to support transition to a recovery identity and targeting beliefs about diets and ‘health’ that are rooted in societal norms.

A key element of recovery was developing a more adaptive relationship with fitness and exercise. This differs from proposed diagnostic criteria for ON, which currently do not include disordered attitudes towards fitness and exercise as a core component [6–8]. One reason for our finding could be due to the representation of more general eating disorder discussions within this recovery space, as evidenced by the findings of the hashtag analysis. Indeed, compulsive exercise often presents as a core feature in recognised EDs such as anorexia nervosa [46], and in our analysis #OrthorexiaRecovery was frequently used alongside hashtags referencing other eating disorders such as anorexia nervosa. However, the significance of fitness and exercise has also been noted in other ON samples [24], in addition to the idealisation of thin and toned bodies [5]. Discussions of exercise may have been so prominent because it is so intimately intertwined with the concept of ‘health’ [47]. In future, researchers could consider further elucidating the relationship between ON, fitness, and exercise. It may be the case that disordered approaches to fitness and compulsive exercise manifest in ON, but attitudes differ when compared with other eating disorders (e.g., compulsive exercise for optimal body functionality, as opposed to/as well as weight loss).

Many of the users discussed the importance of listening to their body, either through ‘intuitive eating’ or ‘intuitive movement’. This reflects the findings of recent research, which has highlighted the value of placing trust in the body for hunger and satiety cues to guide eating [48–50]. While there is currently limited research explicitly looking at the role of intuitive eating in promoting changes in eating behaviour [51], there is evidence that the ability to detect and discriminate interoceptive cues such as hunger and satiety may be impaired in individuals with eating disorders [52, 53]. It is unclear whether such impairment is also present for individuals with ON, and thus much more research is needed to understand how, and whether, therapeutic interventions targeting intuitive eating and exercise might be useful in the context of ON treatment.

Our findings highlight that #OrthorexiaRecovery was generally a supportive space for people with experience of ON, providing a source of network, emotional, and informational support [54]. This provides a counternarrative to much research on ON, which has typically identified social media as a risk factor [15–17]. There is therefore a greater need for more nuanced approaches to studying social media usage within research that focuses on ON and mental health more generally; instead, focusing upon the different types of content users engage with, their reasons for engaging with it, and their frequency of engagement. Nevertheless, we did observe some content which could be triggering for people with ON, similar to Santarossa et al. [23]. Following an analysis of pro-ana and pro-recovery content on Twitter and Tumblr, Branley and Covey [55] recommended that researchers strengthen the presence of positive support sources on public social media platforms or alternatively, improve methods of content moderation. While Branley and Covey [55] focused on eating disorders more generally, similar approaches may also be useful for online recovery communities relating to ON.

### Limitations

Our research is not without limitations. Firstly, as is the case with all research which employs this methodology, we were reliant on users who self-identify with ON and it was not possible to verify their condition. In addition, some posts did not explicitly mention ON, so in some cases it was unclear whether users identified with ON or were using the hashtag to reach the wider eating disorder community. Moreover, the findings of our research are predominantly positive, suggesting that we may not have fully uncovered negative consequences of this online space. In future, qualitative interviews could be conducted with people with ON to further examine their experiences of recovery and the challenges associated with online recovery spaces, and also incorporate operationalised measures of orthorexic traits.

Our research also focused primarily on the textual data uploaded with Instagram posts, meaning the visual data were not analysed in this study. Future analyses of this imagery could yield further insights into conceptualisations of recovery. Our data also only included posts written in English, thus only capturing narratives from a limited number of cultures. In addition, we analysed a relatively small proportion of posts, under a single hashtag, on a single platform. Analysis of data from other platforms and content under other hashtags may provide more nuanced understanding of recovery from ON and could be the subject of future research.

## Conclusion

In conclusion, we found that the #OrthorexiaRecovery Instagram space is being used within a wider context of eating disorder recovery and awareness, but also provides deep insights into experiences of recovery from self-identified ON. The findings suggest that people with self-identified ON experience recovery as a continuous process with many setbacks, and the current invisibility of ON within diagnostic criteria and wider society impedes recovery processes. While working towards recovery, people with self-identified ON aimed to be free from food rules that are rooted in diet culture, become more attuned to their bodies, and develop more adaptive relationships with food and fitness. Users noted a lack of support for people with ON and so used this online space to create a community of support, though some content was observed to be potentially triggering. Our findings highlight the importance of increased recognition of ON and the potential value of targeting societal norms and harnessing social identity resources within therapeutic interventions for ON.

### What is already known

People with ON are using social media such as Instagram to share their experiences of living with the condition. However, there is a lack of research that has harnessed online data to specifically examine recovery from ON.

### What this study adds

The current invisibility of ON within diagnostic criteria and wider society appeared to impede recovery. While working towards recovery, people with ON aimed to be free from diet culture, become more attuned to their bodies, and develop more adaptive relationships with food and fitness. The #OrthorexiaRecovery online space appears to be a supportive community, although some content was potentially triggering for people with ON. Societal norms and social identities may be targets for therapeutic interventions for ON.

## Data Availability

Data are available on request from the corresponding author.
